# Wellbeing in line managers during mandatory working from home: How work and personal factors combine

**DOI:** 10.3389/fpsyg.2022.1041902

**Published:** 2022-12-15

**Authors:** Marco van Gelder, Marc van Veldhoven, Karina van de Voorde

**Affiliations:** Department of Human Resource Studies, Tilburg School of Social and Behavioral Sciences, Tilburg University, Tilburg, Netherlands

**Keywords:** line manager, work related wellbeing, mandatory working from home, COVID-19, workplace innovation

## Abstract

The pandemic, particularly the aspect of forced working from home, has had a major impact on the workforce. Previous studies show that line managers have also experienced severe mental strain during this period. Since it is expected that hybrid working will be more the new normal than the exception in future, this study further examined line managers' work-related wellbeing in terms of engagement and exhaustion. Following the job characteristics model (JCM), we explore the mediating role of meaningful work between workplace innovation before the pandemic and line managers' work-related wellbeing during forced working from home. The underlying idea is that organizations that already adopted workplace innovation practices before the pandemic, give teams and employees more control, thus allowing a more meaningful role for line managers, which positively impacts line managers' work-related wellbeing during the pandemic. In addition, building upon Job Demands-Resources (JD-R) Theory and the role of personal resources therein, we explore digital leadership skills and work–life segmentation preference as moderators between meaningful work and work-related wellbeing. Our findings show that workplace innovation is positively associated with engagement *via* its effect on meaningful work, but not associated with exhaustion. Second, we found that work–life segmentation preference amplifies the relation between meaningful work and engagement (positive link) as well as exhaustion (negative link). This indicates that line managers with a high work–life segmentation preference who have a low score on meaningful work, experience less engagement and more exhaustion than line managers with a high score on meaningful work when working from home. No support was found for the moderation of digital leadership skills in the relationship between meaningful work, engagement, and exhaustion. Based on these results, we discuss implications for research and we provide practice recommendations.

## Introduction

Before COVID-19, working from home was most common among knowledge workers (read: highly educated professionals) and managers whose tasks consist largely of acquiring, disseminating, and/or creating knowledge and information, and for most of them, it pertained only to a small part of their working hours (Parent-Thirion et al., 2017). However, the COVID-19 pandemic created a unique situation in the first half of 2020. In the interest of health, unprecedented measures to stop the spread of the virus (lockdown), including urgent advice/obligations to work at home, have been implemented worldwide. As a result, in 2020, many knowledge workers and managers in the Netherlands were forced to work from home full time. Later in the year, this was temporarily scaled back. However, lockdown and gradual scaling down were repeated during 2021 and 2022 (Ministerie van Algemene Zaken, 2022).

The situation during the lockdowns can be seen as a unique social experiment with compulsory working from home, and this has generated quite a bit of curiosity among psychological researchers (Kniffin et al., 2021). By now, a stream of research has appeared on the impact of forced homeworking on employees' wellbeing and functioning (see Van Veldhoven and Van Gelder, 2020; Ipsen et al., 2021b). However, relatively little attention has hitherto been paid to line managers. Few studies have focused explicitly on this target group (Waizenegger et al., 2020; Kirchner et al., 2021; Teodorovicz et al., 2021; Rodrigues et al., 2022). Waizenegger et al., 2020) investigated how the responsible role of line managers during the mandatory lockdown impacts their wellbeing. These authors found that among this group, mental disorders such as stress and anxiety increased. In a recent study, Rodrigues et al. (2022) reported on the difficulties line managers face in coordinating home-based teams during the pandemic. The main perceived difficulties are (1) performing both personal and professional tasks in the same location; (2) motivating employees in a period when social isolation affects employees' mental health, and (3) keeping team members integrated and within a range of activities in a virtual environment. It is easy to see how such coordination issues may translate into wellbeing issues for line managers themselves.

More research among line managers in the context of forced working from home is important for several reasons. First, like all other employees, they had to adapt their work to the situation, and the question can be asked how they adapted and what impact it had on their functioning and wellbeing. Second, they were responsible for the continuity of work during the mandatory lockdown, with all the complexities attached. Third, they also had a supporting role toward the employees. This often concerned not only a substantive supporting role but also an emotional and mental supporting role. Finally, fourth, after the mandatory lockdown, line managers have an important and even crucial role when it comes to converting the experiences gained during the lockdown into longer term adjustments in the way of working within organizations (Cunningham and Hyman, 1995; Ipsen et al., 2021a; Forbes et al., 2020; Parry et al., 2021).

The above amplifies that research is needed on the health and wellbeing of line managers, particularly during the forced lockdown. For this reason, this study starts from the degree of pre-pandemic workplace innovation, which is the interplay between workplace practices and the participative process and its dual aim of increasing both productivity and quality of working life. The underlying idea is that if organizations have given teams and employees more control in the work context, such organizations would be better able to adapt to changing circumstances (like working from home during lockdowns), which positively impacts line managers' work experiences (i.e., meaningfulness at work) as well as work-related wellbeing (i.e., engagement and exhaustion). Following the job characteristics model, we explore the mediating role of meaningful work between workplace innovation before the pandemic and line managers' work-related wellbeing. Furthermore, building upon Job Demands-Resources Theory and the role of personal resources therein, we explore digital leadership skills and work–life segmentation preference as moderators in the linkage between meaningful work and work-related wellbeing.

## Theory

### Workplace innovation

A growing number of European countries have been developing policy interventions and programs to support companies and their employees in transforming traditional work practices through workplace innovation, typically seeking to achieve a convergence between enhanced business performance and quality of working life (Totterdill, 2015). Workplace innovation is “strategically induced and participatory adopted changes in an organization's practice of managing, organizing and deploying human and non-human resources that lead to simultaneously improved organizational performance and improved quality of working life” (Eeckelaert et al., 2012; p. 6). The basic idea behind workplace-innovation is that neither competitive performance goals on the one hand, nor workplace health and wellbeing on the other hand, can be fully achieved by traditional policy levers such as macro-economic manipulation, skills supply, or health and safety regulations (UKCES, 2009). Workplace innovation is not an end-state, but a dynamic, reflective process where all stakeholders are involved to reflect, learn, and transform work processes and employment processes both to internal and external drivers (Dhondt et al., 2012; p. 2–3).

Four different practices are distinguished within workplace innovation (Oeij et al., 2017; p. 66). (1) *Jobs and teams*. Developing a working environment where employees can develop and deploy competences starts with job design, which assumes that an empowered and self-managed team delivers better performance (Totterdill et al., 2002; Ramstad, 2009; Oeij et al., 2012, 2017). Yet convergence depends on the degree to which structures, systems, industrial relations, and leadership are fully aligned with the empowerment of employees in their day-to-day jobs (Buchanan and Preston, 1992; Boxall, 2003). These interdependencies are explored further in the other three elements. (2) *Organizational structures, management, and procedures*. Jobs and teams should be supported and demonstrate a consistent approach and alignment with the commitment to empowerment and trust (3) *Employee-driven improvement and innovation*. Organizations, where employees have sufficient autonomy to control their work demands, create discretionary room for learning (4) *Co-created leadership and employee voice*. Trust and openness are fundaments in successful collaboration and if expanded between managers, employees lead to information sharing and reduced resistance to change.

The interplay between workplace practices and the participative process is central for workplace innovation and its dual aim of promoting productivity and quality of working life and making an organization more resilient toward change and challenges like the forced lockdown.

### Work-related wellbeing

Work-related wellbeing is one of the aims of workplace innovation. Wellbeing is a term that reflects not only health but also satisfaction with work and life. Wellbeing is a summative concept that characterizes the quality of working life, including aspects of occupational safety and health, and it can be an important determinant of productivity at the individual, firm, and societal levels (Schulte and Vainio, 2010). Different studies on applying workplace innovation practices—composed of the structural and cultural orientation—present evidence on how job autonomy, job flexibility, and participation in organizational life influence the quality of working life and organizational performance (Oeij et al., 2017). Workplace innovation was also connected to “wellbeing at work” in the policy to extend occupational safety and health to “wellbeing at work” [European Agency for Safety Health at Work (EU-OSHA), 2013]. Regarding working from home, in the workplace innovation literature, the home–work interface was already seen as a key working environment determinant of wellbeing at work pre-pandemic (Oeij et al., 2017; p. 114). During the COVID-19 pandemic, several studies were conducted on the wellbeing of employees (Zacher and Rudolph, 2021; Jefferson et al., 2022). These studies have shown an impact on psychological wellbeing, with some respondents experiencing stress, burnout, anxiety, depression, fear of COVID-19, lower job satisfaction, and physical symptoms. This also applies to line managers. Ipsen et al. (2022) states that managers found their work as remote managers more demanding because they worked more hours. Workplace innovation, as implemented in organizations before the pandemic, can be expected to act as a contextual factor enabling line managers to maintain their wellbeing, as control and responsibility are shared on a wider basis within the organization. The pandemic, which one of the external drivers' stakeholders had to deal with, had an impact on the status quo of the way of working. Moving from in-person collaboration and work patterns toward working virtual *via* the work–home interface with a physical disconnect balancing both productivity and work-related wellbeing.

There are many ways to conceptualize wellbeing at work (Taris and Schaufeli, 2014). In this article, we want to investigate a more health-related indicator (emotional exhaustion) and an indicator related to the motivational side of wellbeing (engagement). Emotional exhaustion refers to “feelings of being overburdened and exhausted by the emotional demands of one's job” (Demerouti et al., 2001; p. 2). The degree of engagement can be defined as “a positive, satisfying, work-related state of mind characterized by the dimensions of vitality, dedication, and absorption” (Schaufeli and Bakker, 2004, p. 295).

### The mediating role of meaningful work

In the past, it has been argued in the literature that the experienced meaningfulness of work acts as a crucial mediating factor in translating the impact of workplace innovation antecedents into wellbeing. This is most explicitly found in the job characteristics model (JCM) by Hackman and Oldham (1975).

The job characteristics model (JCM) was developed as a model for job redesign and facilitates workplace intervention, allowing firms to optimize the fit between employees and their jobs (Hackman and Oldham, 1976; Hackman, 1978). Hackman and Oldham (1976) specify five core job characteristics as determinants of various work-related outcomes (skill variation, task identity, task significance, autonomy, and feedback). In the JCM, the effects of task characteristics on work outcomes are mediated by three critical psychological states: (1) perceived meaningfulness, (2) perceived responsibility for outcomes, and (3) knowledge of actual work outcomes. In this study, we only consider the first of these critical psychological states. Reviews of the JCM literature reported some evidence that critical psychological states (like meaningfulness), indeed, mediate between job characteristics and personal outcomes (Fried and Ferris, 1987), but more research is needed.

More recent research points in the direction of a positive influence of the experience of meaningful work on worker wellbeing (Steger, 2012), especially when challenged (like was the case during the pandemic). People who say their work is meaningful and/or serves a social good report better psychological adjustment. People who feel their work is meaningful report greater wellbeing (Arnold et al., 2007). People who feel their work serves a higher purpose also report greater job satisfaction and cohesion in the teams in which they work (Sparks and Schenk, 2001).

In line with the JCM model and recent research on meaningful work, in this study, we, therefore, expect that perceived meaningfulness of work (which we here interpret as a critical psychological state) acts as a mediating variable between workplace innovation before the pandemic and line managers' work-related wellbeing during forced working from home, e.g., high workplace innovation will be associated with high wellbeing *via* high meaningfulness (Hypothesis 1).

### The moderating role of personal resources

Earlier we have argued for the central role of meaningfulness in the link between workplace innovation and wellbeing, using the somewhat older JCM as a starting point. In recent theorizing in work psychology, it is more common to view meaningfulness at work not as a critical psychological state, however, but as a job resource, following Job Demands-Resources (JD-R) Theory (Bakker and Demerouti, 2017). For example, meaningfulness is a job resource that, like other job resources, and positively affects wellbeing.

The Job Demands-Resources model (JD-R) (Demerouti et al., 2001; Bakker and Demerouti, 2014) assumes that employee wellbeing (work engagement and exhaustion) is explained by job demands (workload, time constraints) and job resources (autonomy, social support). Research using JD-R theory has provided evidence for the existence of two simultaneous processes: the health process and the motivational process. High job demands exhaust employees' mental and physical resources and therefore lead to the depletion of energy and to health problems. In contrast, job resources foster employee engagement and extra-role performance. Several studies have shown that job resources may buffer the impact of job demands on stress reactions (Bakker and Demerouti, 2017).

Workplace innovation initiatives entail more control and participatory management methods for teams and employees, and for the line managers, this implies that their work is less characterized by exercising control but rather by managing work based on commitment (Oeij et al., 2017). The management task in such an innovated context is described to be a more meaningful one, more focused on what the organization wants to achieve and/or contribute to society and interacting with employees and teams more equal when compared to a more control-based approach. And such a line management job, rich in the job resource of meaningfulness, is expected to translate into wellbeing at work for line managers. How is such meaningful work and wellbeing maintained during forced working from home, and how might it depend on the person of the line manager?

In the beginning, JD-R studies were mostly restricted to studying how work characteristics interacted toward health and wellbeing, but more recently it has been acknowledged that employees' personal resources can also be important determinants regarding their adaption to work environments (Xanthopoulou et al., 2007). We chose to focus on two person-related characteristics that we think are particularly relevant to investigate in the context of forced working from home. First, we will study the role of work–life segmentation preferences, and second, the role of digital leadership skills.

#### Preference for the segmentation of work and private life

Numerous researchers and professionals have addressed how employees face inter-role conflict, as they are constantly faced with the challenge of juggling their work and private lives (Nippert-Eng, 1996; Ashforth et al., 2000). The theory of boundary management was originally introduced by Christena Nippert-Eng (1996), who states that individuals differ in their preference for setting boundaries between their work and private lives. These boundaries can be seen as a continuum where employees have preferences for either strong and clear or more permeable barriers (Mellner et al., 2014). Nippert-Eng (1996) highlighted two types of preferences among individuals: segmentation and integration. Individuals, who prefer to keep their work and private life separate, thus create two separate segments. These people may, for example, have separate e-mail accounts for work and personal use, avoid using their personal mobile phones for work and engage in work-related phone calls after their working day (Kreiner, 2006; Kreiner et al., 2009). They represent the preference for segmentation.

Forced working from home during the lockdowns represents a situation where those who prefer to keep work and private life apart are challenged more than those who do not have such a preference (Caligiuri and De Cieri, 2021; Fukumura et al., 2021). Furthermore, we argue that this is likely to interact with the level of meaningfulness of the job as experienced by the line managers. When a line manager prefers segmentation, it is easier to see how a line manager accommodates forced working from home when experiencing the line management role as meaningful. We, therefore, expect the positive impact of meaningful work on engagement and its negative impact on exhaustion (that we argued above) to be especially relevant for those line managers who have a segmentation preference. Based on the above, we hypothesize the linkage between meaningfulness as a job resource and wellbeing to be influenced by segmentation preference in such a way that this linkage is stronger in line managers that are high on segmentation preference (Hypothesis 2a).

#### Digital leadership skills

Another personal characteristic that seems highly relevant during the lockdown concerns the degree of digital leadership skills (Zeike et al., 2019). Larjovuori et al. (2016) defined digital leadership as the leaders' ability to create a clear and meaningful vision for the digitization process and the ability to implement strategies to actualize it. To be a successful digital leader, two dimensions of competencies can be distinguished according to Westerman et al. (2012): (1) attitudes, competencies, and behaviors that managers need in the digital age (e.g., digital literacy/competencies) and (2) competencies that help drive digital transformation (e.g., strong leadership skills).

It can be argued that line managers who are high on digital leadership skills find it easier to adapt to their new work context, both in their own working from home experience and in their role as a remote manager toward their team. Meaningfulness at work is thus more easily preserved when the line manager can easily deal with the remote work setting, and this is expected to be easier when digital leadership skills are high. We thus hypothesize that the link between meaningfulness and wellbeing (again, positive for engagement, and negative for exhaustion) is stronger for line managers with high digital leadership skills (Hypothesis 2b).

## Methods

### Procedure of data collection

Convenience sampling was used through the students' and Ph.D. students' networks. Qualtrics was used for the online data collection. An untraceable link was provided per invitee to access the questionnaire and to ensure confidentiality. The questionnaire and cover letter were available in Dutch and English. Data collection took place in April/May 2021. Four inclusion criteria were used. These were determined by four threshold questions in the questionnaire. These threshold questions are: (1) You have worked for this organization for the past 2 years, (2) over the past 2 years, I have had responsibility for at least two direct subordinates, (3) I am a direct subordinate to another manager, and (4) before the COVID-19 pandemic, mainly worked from fixed office locations, and since the COVID-19 pandemic, mainly worked from home.

### Sample

A total of 275 people were approached of whom 52% completed the questionnaire, resulting in 144 respondents. However, some of the respondents indicated that they work less than 4 days per week from home. We decided to, therefore, use as an additional inclusion criterion *post-hoc* that the line managers had to be working from home 4 or 5 days a week at the time of completing the questionnaire. There had to be a forced working from home situation. This brings the final sample to 102.

Within this final sample, 59% identified themselves as male and 40% were female. In total 34% of respondents were between 36 and 45 years old and 34% were between 46 and 55 years old, which is 74% of the total. In total 95% of the respondents indicated that they had completed their higher education (HBO or their master's (WO). In total 30% of the respondents had between 2 and 5 years of service, 31% had between 6 and 10 years of service, and 19% had between 11 and 15 years of service. In total 20% of the sample had more than 15 years of service.

We have compared our sample with national information to assess the representativeness of our sample. According to Statistics Netherlands (CBS Centraal Bureau voor de Statistiek, 2019), 74.6% of managers in the Netherlands are male, which is the majority. In our dataset, there are relatively more female respondents. According to ISBW (ISBW, 2019), 32% of line managers fall into the age group between 36 and 45 years old, and 34% of LM are in the age group between 45 and 55 years old. In our dataset, 34% of line managers fall into the age group between 36 and 45 years old, and 39% of LM are in the age group between 45 and 55 years old which is very much in line with the reference.

### Instruments

Exhaustion was measured using nine items of the QEEW2.0, in need for recovery and detachment from work, with a total of nine items (Van Veldhoven and Meijman, 1994). An example item is “I find it difficult to relax at the end of a working day,” and respondents were asked to “Answer these questions focusing on their current situation.” Respondents could answer using a four-point Likert scale from 1 (never) to 4 (always). Thus, a high score indicates a high degree of exhaustion. The reliability analysis showed good internal consistency (α = 0.90).

Engagement was measured using the ultra-short three-item work engagement scale (UWES-3) reported by Schaufeli and Bakker (2001). A sample item is “At work I burst with energy,” and respondents were asked to answer these questions focusing on their current situation. Respondents could answer on a seven-point Likert scale from 1 (never) to 7 (always). Thus, a high score indicates a high level of engagement. Reliability analysis showed good internal consistency (α = 0.90).

Workplace innovation was measured before the pandemic using the scale developed by Kibowski et al. (2019). The scale consists of a total of 19 items (α = 0.822) and represents all the four domains of workplace innovation that were mentioned earlier in the introduction (e.g., jobs and teams; organizational structures, management, and procedures; employee-driven improvement and innovation; and co-created leadership and employee voice). An example item is: “Before COVID-19, it was highly supported in my department that employees showed personal initiative.” The items were measured by a five-point Likert scale from 1 (strong disagree) to 5 (strong agree). A high score indicates a high level of workplace innovation.

Segmentation preference in relation to work and private life was measured using the scale developed by Kreiner (2006). It has four items. An example item is: “I don't like to think about work while I am home.” The items were measured by a seven-point Likert scale of 1 (strongly disagree) to 7 (strongly agree). A high score indicates a high preference for segmentation. Cronbach's Alpha for the scale is 0.89.

Digital leadership skills were measured using a six-item scale by Zeike et al. (2019). A sample item is “I think using digital tools is fun,” and respondents were asked to answer these questions focusing on their present situation. Respondents could answer using a four-point Likert scale of 1 (completely disagree) to 4 (completely agree). A high score indicates a high level of digital skills. Reliability analysis showed a low, only just acceptable level of internal consistency (α = 0.61). Meaningful work was measured by the Work as Meaning Inventory (WAMI) constructed by Steger (2012). The scale contains 10 items. An example item is “I have found a meaningful career.” A high score means a high level of meaningfulness. Cronbach's Alpha for the scale is α = 0.88.

### Analyses

We first performed a CFA to verify the factor structure of the set of observed variables. Second, we report the means and standard deviations for the scales in the study, and the correlations between the scales. Lastly, the mediation and moderation analyses are presented. The calculations are made in SPSS version 27, AMOS version 27, and Hayes process macro version 4.2. For the mediation, we used Hayes process macro model 4 and for moderation Hayes process macro model 1.

## Results

We have performed a CFA based on the six factors that comprise all the constructs included in the study. For the six-factor model, chi-square = 2,002,800 with degrees of freedom = 1,209. For the one-factor model, chi-square = 3,100,269 with degrees of freedom = 1,224. The results of the six-factor model imply a substantial increase in fit compared to the one-factor model. The chi-square difference is 1,097,469 with 15 degrees of freedom which is highly significant (*p* < 0.001). Based on the reported CFA, we, therefore, conclude that the current study is not overly harmed by common methods bias (e.g., all results boil down to a single method factor because the data all derive from surveys), and that using the six separate scale scores is an adequate approach of processing and interpreting the current data.

Table 1 shows the means, standard deviations, and Pearson correlations (r) between all scales in the study. As expected, the correlation between workplace innovation and meaningful work is significant. The table shows that the relationship between meaningful work and exhaustion is not significant, but the link between meaningful work and engagement is significant. Both personal characteristics, e.g., segmentation preference and digital leadership skills, appear to be unrelated to workplace innovation pre-pandemic and experienced meaningfulness of work, but both appear to be related to the wellbeing measures.

**Table 1:** Descriptive statistics and correlations.

		**M**	**SD**	**MIN**	**MAX**	**1**	**2**	**3**	**4**	**5**	**6**
1	Workplace innovation	3.85	0.40	2.56	4.85	-					
2	Work-life segmentation preference	4.04	1.60	1.50	7.00	−0.04	–				
3	Digital leadership skills	2.91	0.48	2.00	4.00	0.04	−0.21*	-			
4	Meaninful work	3.85	0.56	1.80	5.00	0.31**	−0.13	0.11	-		
5	Exhaustion	2.16	0.59	1.00	4.00	−0.15	0.36**	−0.13	−0.15	-	
6	Engagement	4.83	1.09	1.00	7.00	0.30**	−0.29**	0.20*	0.44**	−0.46**	-

This table shows the means (M), standard deviations (SD), and Pearson correlations (r). Correlation is significant at the 0.05 level (two-tailed).

** Correlation is significant at the 0.01 level (two-tailed).

* *N* = 102.

To determine the mediation effect of meaningful work between workplace innovation and the work-related wellbeing outcomes, we performed two separate mediation analyses using Hayes' process macro number 4, one with engagement and one with exhaustion as the outcome variable.

Table 2A shows that workplace innovation practices are positively associated with meaningful work (B = 0.436, SE = 0.136, *p-*value = 0.002), and meaningful work is positively associated with engagement (B = 0.749, SE = 0.180, *p-*value = 0.000). There is also an indirect effect between workplace innovation and engagement (B = 0.512, SE = 0.257, *p-*value = 0.049).

**Table 2A:** Mediation results for engagement.

**Variable**	**Meaningful work**					**Engagement**				
	**B**	**SE**	***p***	**LLCI**	**ULCI**	**B**	**SE**	***p***	**LLCI**	**ULCI**
Workplace innovation	0.436	0.136	0.002	0.167	0.705	0.512	0.257	0.049	0.003	1.021
Meaningful work	-	-	-	-	-	0.749	0.180	0.000	0.391	1.107

Significant indirect effect with B = 0.326, BootLLCI = 0.014, and BootULCI = 1.021.

Table 2B shows that workplace innovation practices are positively associated with meaningful work (B = 0.436, SE = 0.136, *p-*value = 0.002) but meaningful work is not associated with exhaustion (B = −0.116, SE = 0.109, *p-*value = 0.290). There is no indirect effect between workplace innovation and exhaustion (B = −0.173, SE = 0.155, *p*-value = 0.266).

**Table 2B:** Mediation results for exhaustion.

**Variable**	**Meaningful work**					**Exhaustion**				
	**B**	**SE**	***p***	**LLCI**	**ULCI**	**B**	**SE**	***p***	**LLCI**	**ULCI**
Workplace innovation	0.436	0.136	0.002	0.167	0.705	−0.173	0.155	0.266	−0.481	0.134
Meaningful work	-	-	-	-	-	−0.116	0.109	0.290	−0.331	0.100

Insignificant indirect effect with B = −0.050, BootLLCI = −0.262, and BootULCI = 0.134.

To determine the moderation effect of work–life segmentation preference and digital leadership skills in the relationship between meaningful work and work-related wellbeing outcomes (engagement and exhaustion), we performed four moderation analyses using Hayes' process macro number 1, one for each combination of moderator and outcome.

As shown in Table 3, work–life segmentation preferences strengthen the positive relationship between meaningful work and engagement (B = 0.3135, SE = 0.0843, *p*-value = 0.0003) and become significant with a work–life segmentation preference value of 2.8 and higher. Digital leadership skills fall just short of moderating the relation between meaningfulness and engagement (B = 0.7728, SE = 0.4071, *p*-value = 0.0606). Work–life segmentation preference strengthens the negative relationship between meaningful work and exhaustion (B = −0.1662, SE = 0.0494, *p*-value = 0.0011) and becomes significant with a work–life segmentation preference value of 4.9 and higher. Digital leadership skills do not moderate the relation between meaningful work and exhaustion (B = 0.0005, SE = 0.2491, *p*-value = 0.9984).

**Table 3:** Moderation results.

**Variable**	**B**	**SE**	***t***	***p***	**LLCI**	**ULCI**
Meaningful work > Engagement with Moderator WLSP						
Constant	7.4560	1.5200	4.9053	0.0000	4.4396	10.4724
Meaningful work	−0.5088	0.3866	−1.3159	0.1913	−1.2760	0.2585
Work-life segmentation preference	−1.3620	0.3272	−4.1630	0.0001	−2.0113	−0.7128
Meaningful work × work–life segmentation preference	0.3135	0.0843	3.7166	0.0003	0.1461	0.4809
Meaningful work > Engagement with Moderator DLS						
Constant	9.7890	4.8991	1.9981	0.0485	0.0668	19.5112
Meaningful work	−1.5028	1.2391	−1.2128	0.2281	−3.9618	0.9562
Digital leadership skills	−2.6973	1.6191	−1.6659	0.0989	−5.9103	0.5158
Meaningful work × digital leadership skills	0.7728	0.4071	1.8980	0.0606	−0.0352	1.5807
Meaningful work > Exhaustion with Moderator WLSP						
Constant	−0.6246	0.8896	−0.7021	0.4843	−2.3900	1.1409
Meaningful work	0.5872	0.2263	2.5949	0.0109	0.1381	1.0363
Work-life segmentation preference	0.7635	0.1915	3.9874	0.0001	0.3835	1.1435
Meaningful work × work–life segmentation preference	−0.1662	0.0494	−3.3661	0.0011	−0.2641	−0.0682
Meaningful work > Exhaustion with Moderator DLS						
Constant	3.0943	2.9974	1.0323	0.3045	−2.8540	9.0426
Meaningful work	−0.1420	0.7581	−0.1873	0.8518	−1.6465	1.3625
Digital leadership skills	−0.1366	0.9906	−0.1379	0.8906	−2.1024	1.8292
Meaningful work × digital leadership skills	0.0005	0.2491	0.0020	0.9984	−0.4938	0.4948

## Discussion

First, following the job characteristics model (JCM), we explored the mediating role of meaningful work between workplace innovation before the pandemic and line managers' work-related wellbeing. Our results show that workplace innovation practices are positively associated with meaningful work, and meaningful work is positively associated with engagement. There is also an indirect effect between workplace innovation and engagement. Hence, Hypothesis 1 is confirmed for engagement. These findings are in line with wellbeing as one of the main aims of workplace innovation and recent research that points in the direction of a positive influence of the experience of meaningful work on worker wellbeing (Steger, 2012) especially when challenged (as was the case during the pandemic). On the other hand, our results show that, although workplace innovation practices are positively associated with meaningful work, they are not associated with exhaustion in an indirect way. Hypothesis 1 is, therefore, not confirmed for exhaustion. Several reasons can be given for this lack of confirmation of exhaustion. One of the reasons could be timing. The data collection took place at the relative beginning of the pandemic lockdown. In that period, line managers were maybe too much focused on the business and on keeping the business going and as a consequence their personal wellbeing and exhaustion were not the biggest topic for them at the time. Another explanation could be that the impact of meaningful work on exhaustion depends on personal preferences, and this indeed is what we have tested in the following hypotheses 2a/b.

**Figure 1:**
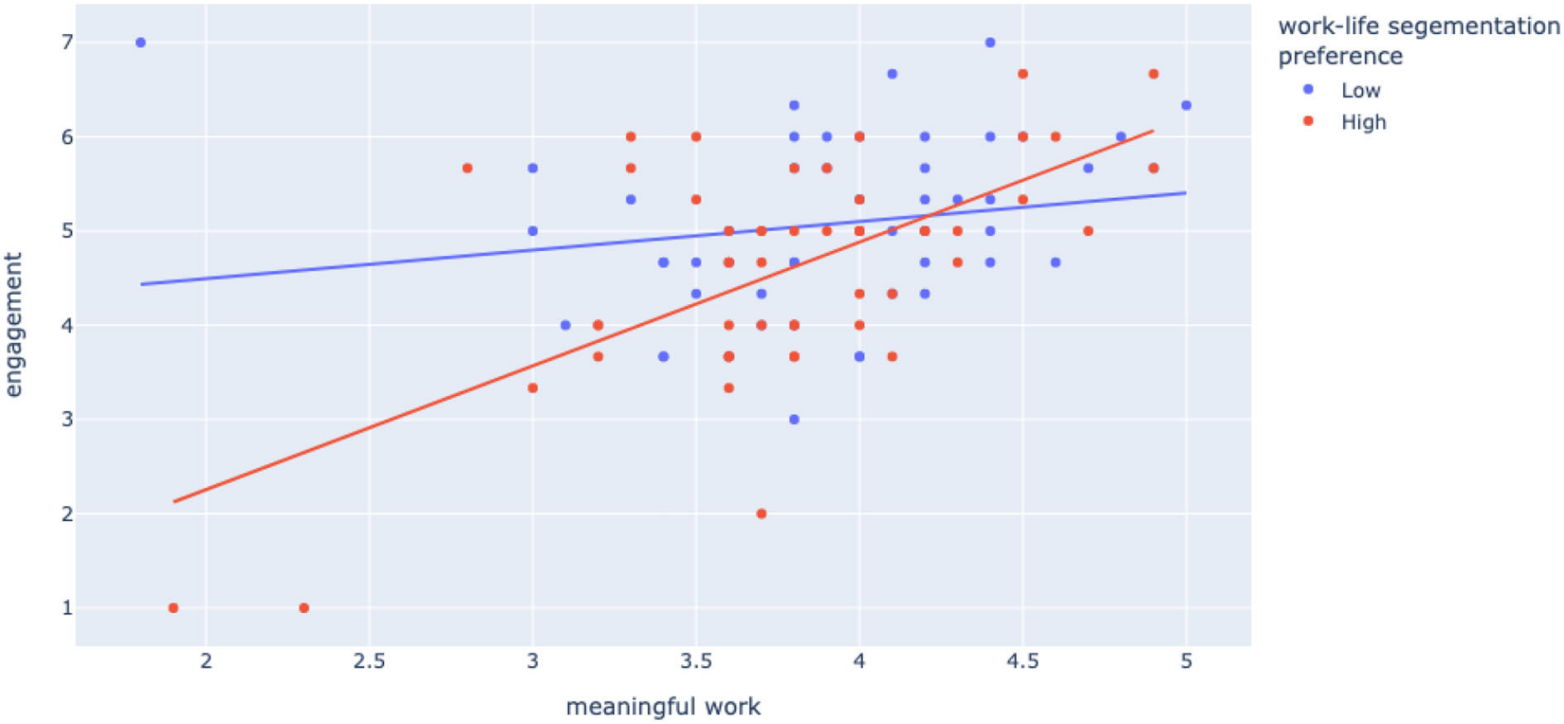
Moderation meaningful work × engagement by work–life segmentation preference.

**Figure 2:**
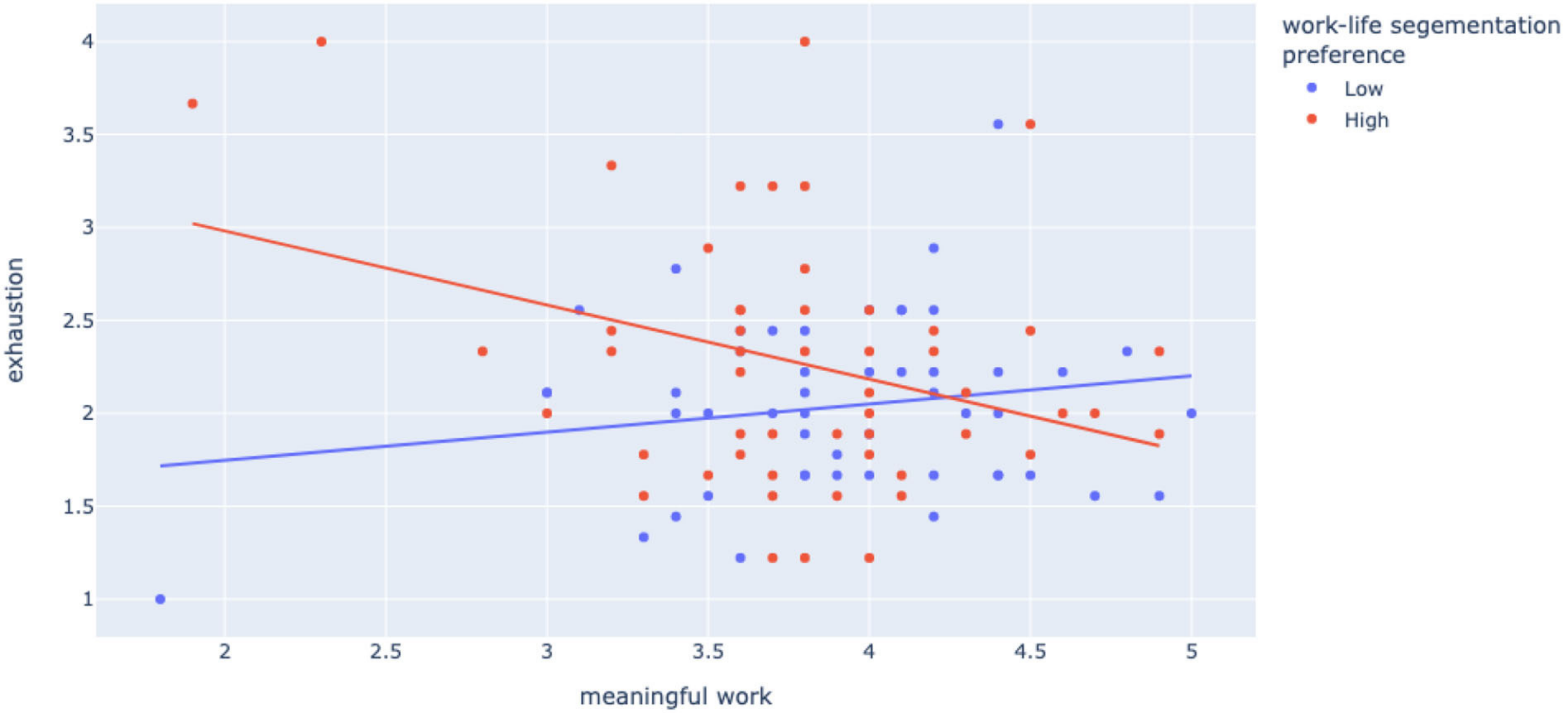
Moderation meaningful work × exhaustion by work–life segmentation preference.

Second, building upon the Job Demands-Resources (JD-R) Theory and the role of personal resources in JD-R Theory, we explored digital leadership skills and work–life segmentation preference as moderators in the link between meaningful work and work-related wellbeing. We expected that the relation between meaningful work and work-related wellbeing would be amplified for those with a high segmentation preference, and this was confirmed for both engagement and exhaustion (Hypothesis 2a). Our results show that work–life segmentation preferences strengthen the relationship between meaningful work and engagement. Work–life segmentation preference also strengthens the negative relationship between meaningful work and exhaustion. When we interpret these findings in combination, we see that—as expected—for line managers with high work–life segmentation preference, work meaningfulness matters in terms of their wellbeing. Earlier we argued and found that workplace innovation is important in creating a management role that can be experienced by managers as meaningful, and now we see how such meaningfulness might translate into wellbeing, depending on the line managers' preferences. Figures 1, 2 further illustrate how work–life segmentation acts as a strong moderator in the link between meaningful work and wellbeing. We can see in the graphs how especially on the low end of meaningfulness scores on wellbeing tend to be unfavorable for high segmentation preference line managers in particular.

We expected that the link between meaningfulness and work-related wellbeing is more easily preserved when the line manager can easily deal with the remote work setting, e.g., when digital leadership skills are high (Hypothesis 2b). Our results, however, do not point toward digital leadership skills being an important moderator in the relation between meaningful work and wellbeing. Hypothesis 2b is, therefore, not confirmed by this study. Several reasons can be given for why we have not found any confirmation for such a role in digital leadership skills. For example, it may be that the IT technology used during the pandemic was not necessarily very different from the technology used and known before; or, the overuse of technology during the pandemic may have changed the response to questions such as “I like using technology,” now being answered to more negatively; finally, it may just be the case that technology as such was a less important position in the work experience of line managers during the pandemic than expected.

### Limitations and strengths, and some recommendations for future research

In this study, we worked with a relatively small sample size (*n* = 102), but relevant to the research question and carefully selected based on several important inclusion criteria. The invitation was performed *via* a snowball sample of the students involved, but we monitored the response rate and relied on known contacts to improve motivation to participate. We checked for the representativeness of our sample for Dutch managers and found that such representativeness is acceptable. The dataset is somewhat skewed in that there are more female respondents than in the national benchmark.

The digital leadership scale is only marginally reliable, and this may have prohibited us from finding the hypothesized moderation for this variable. As the results were close to the significance, it would be important to replicate the current study with a better digital leadership scale. For attrition, we may need to start earlier with more established antecedents such as workload and work–life segmentation preference so that we can better evaluate the (additional) role of workplace innovation. Measuring workplace innovation practices at the individual level has inherent limitations. For our workplace innovation measure, this may imply that it does not capture the complex concept and organizational process of workplace innovation.

Remarkably, we found no direct or indirect effect between workplace innovation and exhaustion and no mediation by meaningfulness in this linkage either, but we found moderation. Personal job resources appear to matter a great deal here, especially segmentation preference. It would be important to study the link between workplace innovation, meaningful work, and exhaustion in further detail with future research.

Finally, we would want to investigate the interactions involved in this study in a different way (e.g., workplace innovation × job demands/resources) by analyzing the possibility of the existence of multiple configurations of antecedents that may cause exhaustion and/or engagement, e.g., by using a configurational analysis method like fsQCA (Ragin, 2000).

### Practice recommendations

Based on this study we claim that line managers who work in an environment with a high degree of workplace innovation established before the pandemic experienced their work as more meaningful during the pandemic and were more engaged and less exhausted. Moving forward, we see that hybrid working is becoming more and more the new normal, and we expect line managers to play an important role in the success of such hybrid working. It is, therefore, advisable for organizations to invest more in workplace innovation to ensure meaningful work and wellbeing in line managers as core players in the transition toward hybrid work.

Hybrid working implies that work will take place more independently of time and place and this may be at odds with line managers' preferences as to work–life segmentation. The underlying risk is that work and private time are increasingly mixed causing a negative effect on line managers wellbeing for those who like to keep things separated. In this research, we have seen that the degree of work–life segmentation preference, indeed, strengthens the link between meaningful work on engagement (positive) as well as exhaustion (negative). It is, therefore, recommended that organizations pay attention to work–life segmentation preferences among line managers especially when workplace innovation in the organization is not yet so advanced and/or a line management role that is experienced as meaningful is not yet possible. In such circumstances, line managers that prefer segmentation are experiencing low engagement and high exhaustion. Investing in workplace innovation practices is a direction for moving forward in such settings as elsewhere, but until then it is important to understand that a more control-oriented leadership role is difficult to achieve from a distance, especially if you prefer work to be work and home to be home.

## Conclusion

We can conclude that line managers who reported that their organization was already practicing workplace innovation before the pandemic reported higher experienced meaningfulness in their work currently, as well as higher work engagement in connection with this. Workplace innovation should, therefore, be encouraged, also as a strategy for enabling line managers in their work. Furthermore, the degree of work–life segmentation preference strengthens the positive effect of meaningful work on engagement and strengthens the negative effect on exhaustion and deserves more attention in this important organizational group when considering the transition to hybrid working as the new normal.

## Author's note

This cross-sectional questionnaire study was performed as part of a larger research project in which three master students (Rinne van Krieken, Nick van de Kerkhof, and Justyna Michalik) and Ph.D. student Marco van Gelder from Tilburg University collaborated under the supervision of Marc van Veldhoven. Special thanks to Rinne, Nick and Justyna for their efforts. A special thanks also to Franciscus Martinus “Rob” Middendorp (Veldhoen + Company) for his contribution to data processing and analysis.

## Data availability statement

The raw data supporting the conclusions of this article will be made available by the authors, without undue reservation to any qualified researcher.

## Author contributions

MvG, MvV, and KvdV contributed to the conceptualization, design, and conduct of the study. All authors contributed to writing the manuscript and doing the analyses.
